# Exploring Material Properties and Device Output Performance of a Miniaturized Flexible Thermoelectric Generator Using Scalable Synthesis of Bi_2_Se_3_ Nanoflakes

**DOI:** 10.3390/nano13131937

**Published:** 2023-06-26

**Authors:** Zicheng Yuan, Xueke Zhao, Canhui Wang, Shuang Hang, Mengyao Li, Yu Liu

**Affiliations:** 1 Reactor Engineering Sub-Institute, Nuclear Power Institute of China, Chengdu 610213, China; yuanzc_npic@163.com (Z.Y.); crazydale@163.com (C.W.); 2 School of Physics and Microelectronics, Zhengzhou University, Zhengzhou 450052, China; xuekezhao12@163.com; 3 Inter-University Institute for High Energies, Université Libre de Bruxelles, 1050 Brussels, Belgium; hangshuang1991@163.com; 4 School of Chemistry and Chemical Engineering, Hefei University of Technology, Hefei 230009, China

**Keywords:** Bi_2_Se_3_, energy conversion, thermoelectric devices

## Abstract

Environmental heat-to-electric energy conversion presents a promising solution for powering sensors in wearable and portable devices. However, the availability of near-room temperature thermoelectric (TE) materials is highly limited, posing a significant challenge in this field. Bi_2_Se_3_, as a room-temperature TE material, has attracted much attention. Here, we demonstrate a large-scale synthesis of Bi_2_Se_3_ nanoflakes used for the microflexible TE generator. A high-performance micro-TE generator module, utilizing a flexible printed circuit, has been designed and fabricated through the process of screen printing. The TE generator configuration comprises five pairs of PN TE legs. The p-type TE leg utilizes commercially available Sb_2_Te_3_ powder, while the n-type TE leg employs Bi_2_Se_3_ nanoflakes synthesized in this study. For comparative purposes, we also incorporate commercially available Bi_2_Se_3_ powder as an alternative n-type TE leg. The optimal performance of the single-layer microflexible TE generator, employing Bi_2_Se_3_ nanoflakes as the active material, is achieved when operating at a temperature differential of 109.5 K, the open-circuit voltage (*V*_OC_) is 0.11 V, the short circuit current (*I*_SC_) is 0.34 mA, and the maximum output power (*P*_MAX_) is 9.5 μW, much higher than the generator consisting of commercial Bi_2_Se_3_ powder, which is expected to provide an energy supply for flexible electronic devices.

## 1. Introduction

Thermoelectric (TE) materials possess the ability to convert heat into electricity, presenting a promising avenue to address the energy crisis and environmental challenges stemming from the overconsumption of conventional fossil fuels [1,2,3]. In particular, as the demand for portable, wearable, and self-powered electronics continues to rise, flexible TE generators have emerged as a promising power source due to their ability to harness waste heat, which is abundantly available [4]. The energy-conversion efficiency of a (TE) material is quantified using the dimensionless figure of merit, ZT = *S^2^σT*/*κ*, where S represents the Seebeck coefficient, σ denotes the electrical conductivity, κ signifies the thermal conductivity, and T corresponds to the absolute temperature. The power factor (PF ≡ *S^2^σ*) in the numerator represents the electronic energy-conversion capability of the TE material. On the other hand, the denominator (*κ = κ_L_ + κ_e_*), which is the sum of the lattice thermal conductivity (κ_L_) and electronic thermal conductivity (κ_e_), quantifies heat leakage in the system.

Due to the rapid advancement of TE materials, TE devices have garnered significant attention in recent years due to their immense potential for applications in green energy and energy management [5]. Based on their specific applications, TE devices can be categorized into two main types: TE coolers (TECs) and TE generators (TEGs). TEGs harness the Seebeck effect to convert heat into electricity, thereby offering significant potential for solar energy utilization and waste heat recovery from various sources such as fuel cells, industrial dryers, automobile exhaust systems, and refuse burning [6,7,8]. The TE cooler (TEC) operates by utilizing the Peltier effect, where electricity is employed to transfer heat to high-temperature heat sources. This characteristic has led to the widespread application of TECs in cooling, refrigeration, and temperature-control technologies [7]. TEG technology is regarded as one of the primary methods for directly generating electrical current from a temperature difference [9].

Flexible TEGs have attracted significant research interest due to their importance in enabling wearable, self-powered mobile electronics. The successful implementation of flexible TEGs relies on achieving both high mechanical and electrical stability when subjected to repeated tensile and compressive strains [6,10,11,12,13,14,15,16]. One of the key considerations lies in the design of external substrates that accommodate tens or hundreds of TE legs. These substrates need to possess flexibility for wearable applications while also exhibiting high thermal tolerance to withstand extreme conditions during the fabrication process. Furthermore, it is essential to minimize thermal-energy loss across the substrate in order to enhance the energy-conversion efficiency of the TEG when operating on a heat source. The submicron morphology characteristics of film thermoelectric materials enable them to exhibit intriguing performance and extend their applications. These film thermoelectric materials are manufactured on flexible substrates using various advanced processes, resulting in the development of film thermoelectric modules and film thermoelectric detectors [17].

This study builds upon previous fabrication techniques of flexible thermoelectric devices and aims to develop a novel nanoscale printing conductive ink based on Bi_2_Se_3_.In the realm of conductive inkjet printing inks, flake-shaped powders serve as conductive fillers and exhibit favorable characteristics in terms of lateral conductivity. Simultaneously, they contribute to an increased effective length of thermal-conduction pathways. Therefore, this work aims to prepare nanoscale flake-shaped Bi2Se3 powder as a conductive filler for printing ink through a hydrothermal synthesis method.

In our approach to developing a microflexible TEG, we utilize Bi_2_Se_3_ nanoflakes as the n-type TE material. Bi_2_Se_3_ is a widely studied representative material that operates within the room-temperature range, featuring a bandgap of 0.3 eV [18,19,20,21,22,23,24,25,26,27,28,29,30,31,32,33,34,35,36]. For the p-type TE legs in our microflexible TEG, we utilize commercially available Sb_2_Te_3_ powders. The TEG is composed of five pairs of PN TE legs, where p-type and n-type rectangular TE legs are printed in succession onto a polyimide substrate. Gold-plated copper-foil electrodes are employed to establish the necessary electrical connections. Our microflexible TEG demonstrates an impressive output power of 9.5 μW when operating under a temperature difference of 109.5 K.

## 2. Experimental

***Chemicals and solvents:*** Bismuth (III) nitrate pentahydrate (Bi(NO_3_)_3_·5H_2_O, ≥99.99%) and polyvinylpyrrolidone (PVP, (C_6_H_9_NO)n, average mol wt ~55,000), were procured from Sigma Aldrich and used without further purification. Sodium selenite (Na_2_SeO_3_, ≥98%), potassium hydroxide (KOH, ≥98%), and ethylene glycol (EG, HOCH_2_CH_2_OH, 99%) were obtained from Fisher. Analytical-grade acetone and ethanol were obtained from various sources. All chemicals and solvents were used as received in their original form. The syntheses were conducted using a standard vacuum/dry argon Schlenk line.

***Synthesis of Bi*_2_*Se*_3_ *Nanoflakes:*** The synthesis of Bi_2_Se_3_ nanoflakes was conducted according to a procedure previously described by Li et al. [37]. In a typical synthesis, 5 mmol of Bi2Se3 was prepared by dissolving 10 mmol of Bi(NO_3_)_3_·5H_2_O, 15 mmol of Na_2_SeO_3_, 50 mmol of KOH, and 0.5 g of PVP in 200 mL of ethylene glycol (EG) within a 500 mL three-neck flask. The mixture was stirred under an argon atmosphere at room temperature for 30 min. Subsequently, the solution was heated to 180 °C, and during the process, the color of the solution gradually changed from slightly brown to black around 160 °C. The reaction mixture was maintained at 180 °C for 3 h. After the reaction time, the solution was naturally cooled to room temperature by removing the heating mantle. Nanoflakes were purified by centrifugation. In the first step, the solid product was obtained by introducing acetone to the solution followed by centrifugation. Subsequently, ethanol was employed to disperse the nanoflakes, and acetone was used for their reprecipitation. This process was repeated twice. Finally, the nanoflakes were dried under vacuum at room temperature. The synthesis protocol was optimized to yield 3 g of nanoflakes per batch.

***Bulk nanomaterial forming:*** The dried Bi_2_Se_3_ nanoflakes were subjected to annealing at 350 °C for 60 min under an argon flow inside a tube furnace. Following the annealing process, the powder was loaded into a graphite die and compacted into cylindrical shapes (Ø 10 mm × 10 mm) using a custom-made hot press. The hot-press temperature was set at 485 °C, and the pressure was gradually increased to 80 MPa over a duration of 4 min. The relative densities of the compacted pellets were determined using the Archimedes’ method, revealing values of approximately 92% of the theoretical density. Subsequently, the cylinders were cut in two perpendicular directions: along the pressing direction and within the plane of the cylinder. These cuts were made to facilitate the measurement of the TE properties [38,39].

***TE generator design:*** The flexible TEG consists of five pairs of PN TE legs. The P-type TE leg is commercial Sb_2_Te_3_ powder, and the Bi_2_Se_3_ nanoflakes synthesized in this work are used as the N-type TE leg. As a contrast, commercial Bi_2_Se_3_ powder is also used as an N-type TE leg for the flexible TEG by screen printing. The TE ink binder mixture consisted of various components. The polymer binder used was PPGDGE/D.E.R.736 (polypropylene glycol diglycidyl ether epoxy resin, Sigma-Aldrich LLC, Singapore). The hardener employed was 4-MHHPA (methylhexahydrophthalic anhydride, Sigma-Aldrich LLC), while the catalyst used was 2E4MZ-CN (1-cyanoethyl-2-ethyl-4-methylimidazole, Shikoku Chemicals Corporation). As a nonreactive diluent, butyl acetate (Sigma-Aldrich LLC) was incorporated. The epoxy-to-hardener equivalent weight ratio was maintained at 1:0.85. Additionally, a catalyst concentration of 0.5 wt% was utilized in the system. Butyl acetate was added during the printing process as needed to adjust the viscosity of the ink. P-type and N-type rectangular TE legs are printed successively on a polyimide substrate with gold-plated copper-foil electrodes. The TE ink was subjected to sintering within a tube furnace under a nitrogen (N2) atmosphere. The heating rate employed was 4 °C/min, and the temperature was held at 275 °C for a duration of 3 h [40,41]. Additionally, standard samples measuring 5 mm × 15 mm and 17 mm × 17 mm were printed for the purpose of measuring the TE properties, as well as the charge carrier concentration and mobility of the film, respectively.

***Microstructural and chemical characterization:*** X-ray diffraction (XRD) analyses were conducted using a Bruker AXS D8 ADVANCE X-ray diffractometer with Cu-Kα radiation (λ = 1.5406 Å). The 2θ angle range was set from 5° to 80°, and the scanning rate was 4°/min. The size and morphology of nanoparticles were examined using field-emission scanning electron microscopy (SEM) on an Auriga Zeiss microscope operated at 5.0 kV. The material composition was analyzed using an Oxford energy dispersive X-ray spectrometer (EDX) attached to a Zeiss Auriga SEM, operating at 20.0 kV. The crystallographic structure and chemical composition were analyzed by high-resolution transmission electron microscopy (HRTEM) using a Tecnai F20 field-emission gun microscope operated at 200 keV, equipped with an embedded Gatan QUANTUM image filter. X-ray photoelectron spectroscopy (XPS) measurements were performed on a Specs system (Specs GmbH, Berlin, Germany) equipped with a Mg anode XR50 source operating at 250 W and a Phoibos 150 MCD-9 detector (Specs GmbH, Berlin, Germany). The pressure in the analysis chamber was maintained below 10^−7^ Pa. Data processing was carried out using the CasaXPS program (Casa Software Ltd., Devon UK).

***Thermoelectric characterization of bulk materials:*** Seebeck coefficients were measured using a static DC method, while the electrical resistivity data were obtained using a standard four-probe method. Both measurements were conducted simultaneously using an LSR-3 LINSEIS system under a helium atmosphere. The temperature range for the measurements was from room temperature to 623 K. Each sample was measured at least three times during the heating process up to approximately 623 K. To account for the system accuracy and measurement precision, an estimated error of approximately 4% was considered for both the electrical conductivity and Seebeck coefficient measurements. The thermal conductivities (κ_total_) were determined by multiplying the thermal diffusivity (λ), the constant-pressure heat capacity (C_p_), and the density of the material (ρ). The thermal diffusivities of the samples were determined using a Xenon Flash Apparatus XFA600, with an estimated error of approximately 5%. The constant-pressure heat capacity (C_p_) was estimated using empirical formulas based on the Dulong–Petit limit (3R law). The density values were measured using the Archimedes’ method. To maintain clarity in the figures, error bars were not included.

***Performance Characterization of Film and Flexible TEG***: The electrical resistivity and Seebeck coefficient measurements were conducted using a commercial system (CTA-3, Cryoall) in the temperature range from room temperature to 250 °C. The carrier concentration and mobility were determined using a Hall measurement system (HT-50, HONOR TOP) employing the Van der Pauw method. The measurements were performed under a magnetic field of 0.3 T and a current of 10 mA. To monitor the temperature during the tests, four J-type thermocouples were positioned at the center, side, hot end, and cold end of the device. The I–V characteristics were scanned and measured using a semiconductor parameter analyzer (Keithley 4200-SCS).

## 3. Results and Discussion

### 3.1. Nanomaterial

**Figure 1:**
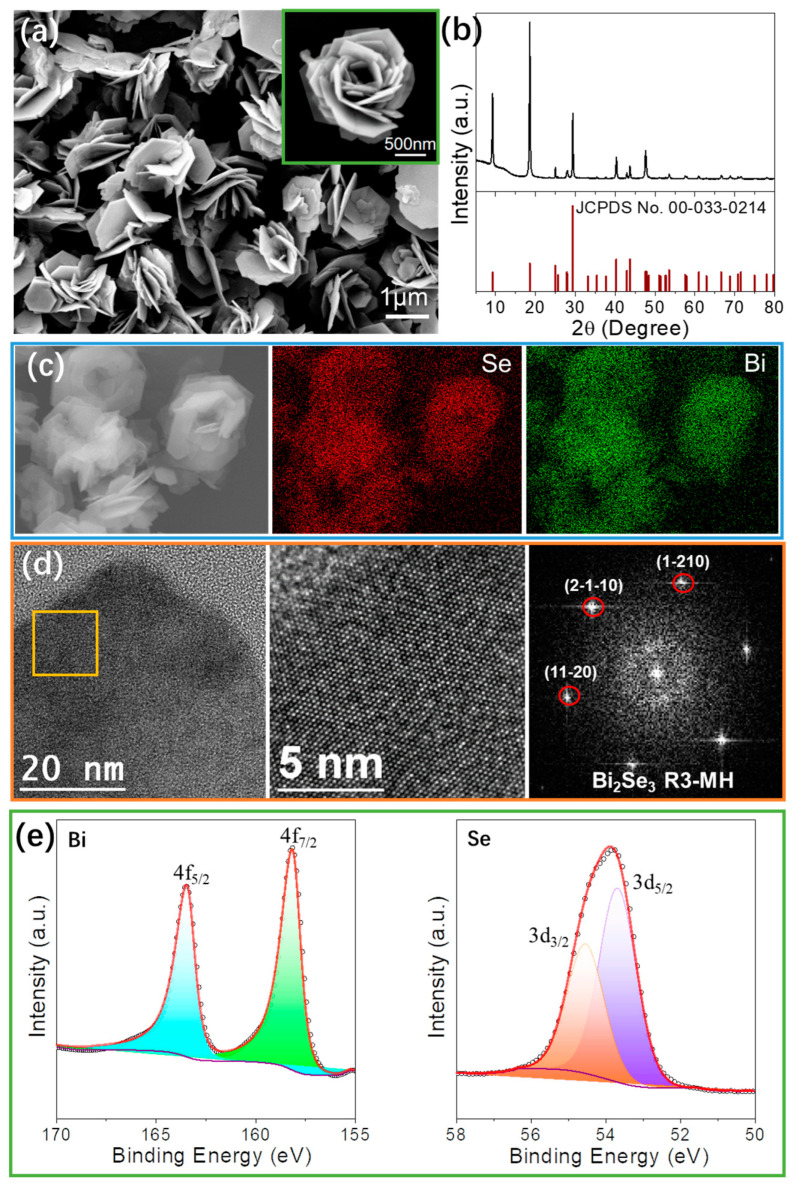
(**a**) SEM micrograph, (**b**) XRD pattern, and (**c**) EDX mapping of Bi_2_Se_3_ nanoflakes; (**d**) HRTEM micrograph, detail of the orange squared region and its corresponding power spectrum; (**e**) Bi 4f and Se 3d high-resolution XPS spectra obtained from Bi_2_Se_3_ nanoflakes.

Figure 1a shows a SEM micrograph of the flower-like Bi_2_Se_3_ nanoflakes obtained following the above synthesis method. Figure 1b displays the X-ray diffraction (XRD) analysis results, which confirmed that the crystal structure of the synthesized nanoflakes corresponded to the rhombohedral Bi_2_Se_3_ phase (JCPDS No. 00-033-0214). The EDX elemental composition maps obtained from the left micrograph are presented in Figure 1c. As can be seen, the maps reveal that the nanoparticles are mainly composed of Bi and Se. Both distribute in a uniform way. HRTEM micrographs of the Bi_2_Se_3_ samples are shown in Figure 1d, Bi_2_Se_3_ nanoflakes show good crystallinity. The orange squared region in the micrograph provides further details of interest. The corresponding power spectrum reveals that this nanoparticle exhibits a crystal phase that is potentially consistent with the hexagonal phase of Bi_2_Se_3_ (space group = R3-MH). The lattice parameters were determined to be a = b = 4.1340 Å and c = 28.6300 Å. Additionally, from the analysis of the crystalline domain, the measured lattice fringe distances within the Bi_2_Se_3_ structure were found to be 0.205 nm, 0.205 nm, and 0.204 nm at angles of 60.96° and 120.86°. These measurements are indicative of the hexagonal Bi_2_Se_3_ phase, visualized along its [0001] zone axis. The valence analysis of elements for the Bi2Se3 nanoflakes was performed using XPS characterization, as shown in Figure 1e. The Bi 4f spectrum exhibited a perfect fit with two peaks at binding energies of 163.4 eV and 158.1 eV, corresponding to Bi 4f_5/2_ and 4f_7/2_, respectively. These binding energies are indicative of the valence state in the Bi_2_Se_3_ compound. Similarly, the Se 3d spectrum displayed a perfect fit with two peaks for Se 3d_3/2_ and 3d_5/2_ at binding energies of 54.5 eV and 53.7 eV, respectively. These results confirm that the synthesized Bi_2_Se_3_ nanoflakes possess a valence state of -2 for Se and +3 for Bi [42,43].

### 3.2. Consolidation of Bi_2_Se_3_ Nanoflakes

The Bi_2_Se_3_ nanoflakes underwent an annealing process at 350 °C for 60 min under an argon flow in a tube furnace. Subsequently, they were subjected to hot pressing under an inert atmosphere to form cylindrical pellets with dimensions of 10 mm in diameter and 10 mm in height. The hot-pressing temperature was set to 480 °C, and a pressure of 80 MPa was applied for 4 min. The resulting cylinders were then cut in two directions: along the pressing direction (//) and within the cylinder plane (⊥), which were used for measuring the thermoelectric (TE) properties (refer to Figure S1). The hot-pressing and cutting processes led to cylinders with relative densities of approximately 93% of the theoretical value, as determined by the Archimedes’ method.

**Figure 2:**
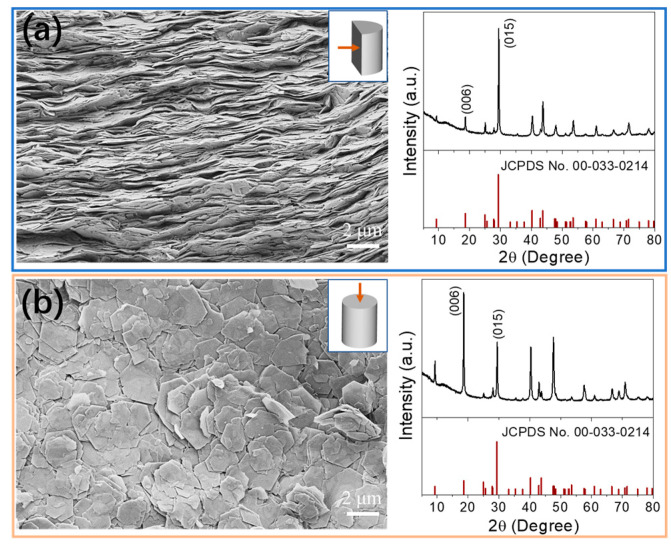
(**a**) Cross-section SEM micrograph and XRD pattern of Bi_2_Se_3_ pellet; (**b**) Top-view SEM micrograph and XRD pattern of Bi_2_Se_3_ pellet.

Figure 2 presents top-view and cross-section scanning electron microscopy (SEM) images of the consolidated Bi_2_Se_3_ pellets, revealing a distinct preferential orientation. The layered structures within the pellets are clearly observed, perpendicular to the direction of pressure, indicating their suitability for flexible thermoelectric generators (TEGs). X-ray diffraction (XRD) analysis of the cylindrical pellets, performed in two perpendicular directions, with the diffraction plane normal and parallel to the pressing direction, confirms the strong preferential orientation of Bi_2_Se_3_ crystal domains within the pellets. The intensity of XRD peaks differs between the two directions, particularly for the lattice planes of [006] and [015], which can be attributed to the variation in crystallographic planes after the hot-pressing process in the two directions.

**Figure 3:**
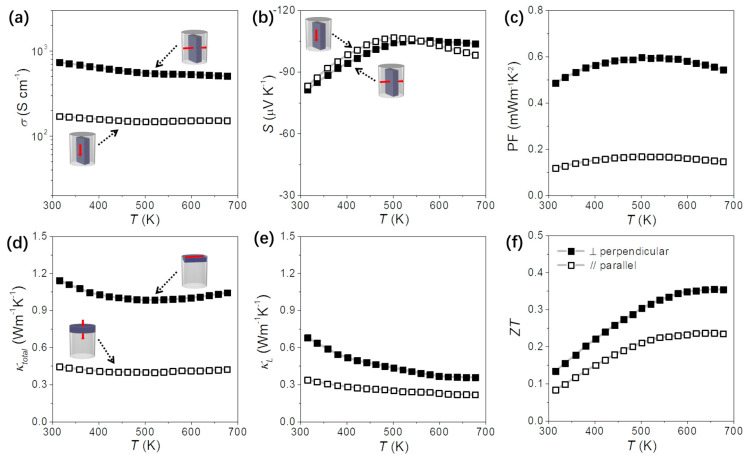
TE properties of Bi_2_Se_3_ nanomaterials measured along two directions of the pressure axis: (**a**) electrical conductivity, σ; (**b**) Seebeck coefficient, S; (**c**) power factor, PF; (**d**) total thermal conductivity, κ; (**e**) thermal conductivity after subtraction of electronic component, κ–κ_e_; and (**f**) TE figure of merit, ZT.

Figure 3 displays the electrical conductivity (σ), Seebeck coefficient (S), power factor (PF), total thermal conductivity (κ), lattice conductivity (κ_L_), and the TE figure of merit (ZT) of the Bi_2_Se_3_ pellets obtained from the two normal directions. As expected, σ_⊥_ is much higher than σ_//_ due to the higher charge carrier mobilities in the *bc* crystal plane compared with *a* direction and the extended size of the crystal domains in the ⊥ direction within the layered pellets. Meanwhile, *S* values are the same in both directions. These two variables result in a higher power factor in the vertical direction of the sample. In addition, κ_⊥_ is much higher than κ_//_ due to the higher intrinsic κ in the *bc* plane and to the extended size of the crystal domains in the plane ⊥ to the press axis. Overall, the ZT values of the Bi_2_Se_3_ vertical to the pressing direction are about 0.35, which is higher than along the pressing direction.

Figure 3 depicts the electrical conductivity (σ), Seebeck coefficient (S), power factor (PF), total thermal conductivity (κ), lattice conductivity (κ_L_), and the thermoelectric figure of merit (ZT) of the Bi_2_Se_3_ pellets obtained from the two normal directions. As anticipated, the electrical conductivity in the perpendicular direction (σ_⊥_) is significantly higher than that in the parallel direction (σ_//_). This difference arises from the higher charge carrier mobilities in the crystal plane perpendicular to the pressing direction (bc plane), as well as the larger size of the crystal domains in the perpendicular direction within the layered pellets. On the other hand, the Seebeck coefficient (S) values are consistent in both directions. These two factors contribute to a higher power factor (PF) in the vertical direction of the sample. Furthermore, the total thermal conductivity in the perpendicular direction (κ_⊥_) is considerably higher than that in the parallel direction (κ_//_), primarily due to the higher intrinsic thermal conductivity (κ_L_) in the bc plane and the larger size of the crystal domains in the plane perpendicular to the pressing axis. Overall, the figure of merit (ZT) values for the Bi_2_Se_3_ pellets in the vertical direction relative to the pressing direction are approximately 0.35, which is higher than that along the pressing direction.

### 3.3. Flexible TE Generator

**Figure 4:**
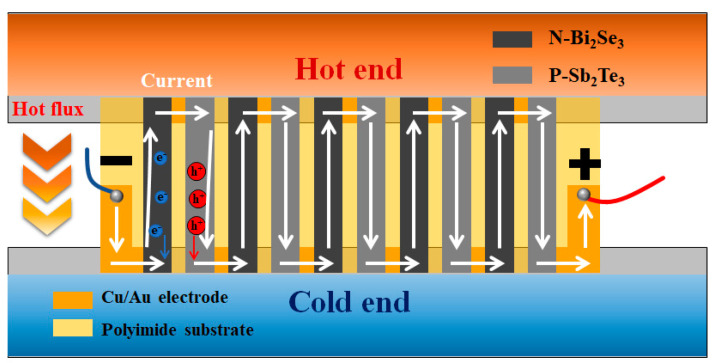
Conceptual design and principal model of the flexible TEG.

Figure 4 is the conceptual design and principal model of the flexible TEG. The module of the flexible thermoelectric generator (TEG) comprises several components, including a polyimide substrate, connecting electrodes, and both n-type and p-type thermoelectric (TE) legs. The p-type TE legs used by commercial Sb_2_Te_3_ powder, and the Bi_2_Se_3_ nanoflakes synthesized in this work are used as the n-type TE legs. As a contrast, commercial Bi_2_Se_3_ powder is also used as an n-type TE leg for the flexible TEG by screen printing. The single-layer module consists of a total of five n-type and five p-type legs. When a temperature difference is applied, it induces the movement of hole carriers and electron carriers within the semiconductor material, causing them to flow from the hot side to the cold side. By connecting the TE legs in series, the spatial potential of the single-layer module is increased, allowing for enhanced thermoelectric performance. The rectangular TE legs are printed successively on a polyimide substrate with gold-plated copper foil electrodes. The TE ink was subjected to a sintering process under a nitrogen (N_2_) atmosphere using a tube furnace. The heating rate during the process was set to 4 °C/min, and the temperature was maintained at 275 °C for a duration of 3 h. This controlled sintering process helps in enhancing the material properties and consolidating the TE ink into a more stable and functional form. After the relevant process, the flexible TEGs were shown in Figure S2. The parameters of the flexible TEG are shown in Table S1. The thickness of the commercial Sb_2_Te_3_ and commercial Bi_2_Se_3_ are about 60 μm, and the thickness of the Bi_2_Se_3_ nanoflakes is 8 μm, which is much thinner than commercial and more beneficial to the application of microflexible TEG.

**Figure 5:**
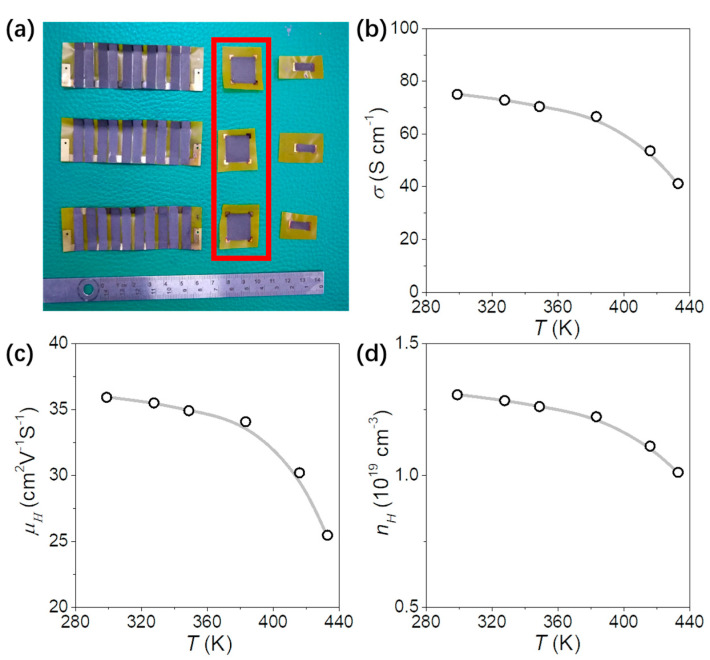
(**a**) Photograph of the flexible TEG and TE films. (**b**) electrical conductivity, σ; (**c**) mobility, μ_H_; and (**d**) carrier concentration, n_H_ of Bi_2_Se_3_ film.

The TE inks were utilized to produce printed TE thick films with dimensions of 5 mm × 15 mm and 17 mm × 17 mm. These films were subsequently employed to measure the TE properties as well as the charge carrier concentration and mobility of the materials. The corresponding results are presented in Figure 5a. The square samples are used to measure the hall effect. The carrier mobility and carrier concentration are decreased and correlated with temperature in the temperature range of 300~430 K. The mobility of Bi_2_Se_3_ nanoflakes printed film decreased from 35.9 cm^3^·V^−1^·s^−1^ to 25.5 cm^3^·V^−1^·s^−1^, and its carrier concentration decreased from 1.31 × 10^19^ cm^3^ to 1.01 × 10^19^ cm^3^. The carrier mobility and carrier concentration of Sb_2_Te_3_ powder were shown in Figure S3. The mobility of the Bi_2_Se_3_ film is higher than Sb_2_Te_3_ film; this is due to the Bi_2_Se_3_ nanoflakes introducing a huge specific surface area, which makes the material bonding strength excellent after curing. According to Equation (1), the above two semiconductor parameters jointly determine the conductivity of the material, which makes the conductivity and temperature also have a negative correlation, from 75.0 S·cm^−1^ to 41.2 S·cm^−1^ in the temperature range of 300~430 K.

(1) σ=neμ

(2) $$S=\frac{8\pi^{2}k_{B}{}^{2}}{3eh^{2}}m*T{\left(\frac{\pi }{3n}\right)}^{2/3}$$

**Figure 6:**
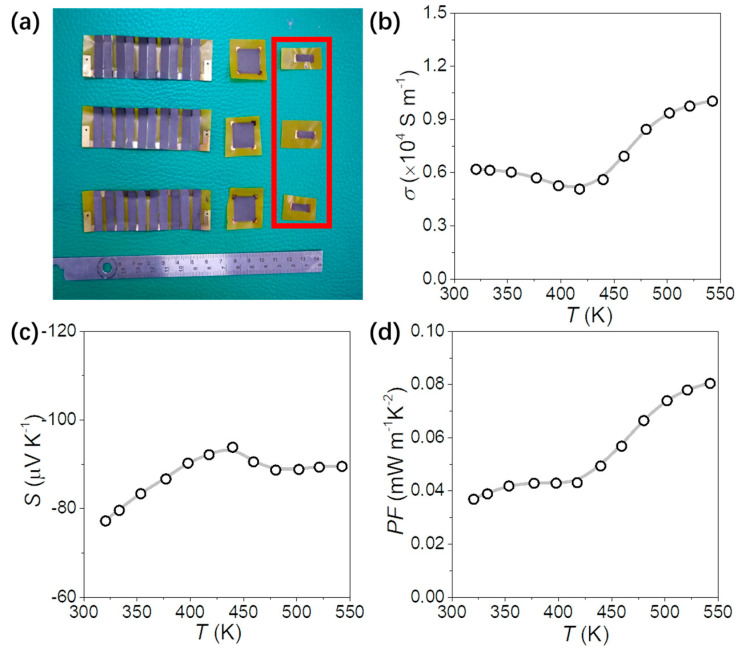
(**a**) Photograph of the flexible TEG and TE films; (**b**) electrical conductivity, σ; (**c**) Seebeck coefficient, S; and (**d**) power factor, PF of Bi_2_Se_3_ film.

The rectangular TE films are also used for the Seebeck performance test, shown in Figure 6 and Figure S4. The temperature dependence of the Seebeck coefficient of the Bi_2_Se_3_ nanoflakes film is similar to that of the bulk material. According to Equation (2), the Seebeck coefficient is negatively correlated with the carrier concentration, combined with the temperature dependence of Figure 5d and Figure 6c from room temperature to 450 K; the relationship between the two is in line with this theory. Figure 5b shows the electrical conductivity measured by the Van der Pauw method and Figure 6b shows the electrical conductivity measured by the straight four-point probe method. From room temperature to 450 K, the change trend of the two is highly consistent, which proves that the printed TE material is in the plane direction. The electrical transport properties are symmetric and uniformly distributed. The *σ* of the printed film is much lower than the bulk materials due to the lower density of the film materials, which leads to the lower PF compared with the bulk materials. The performance of matrix thermoelectric materials in thick-film formulations produced by resin ink screen printing may exhibit some differences compared to bulk materials. The electrical conductivity of the printed samples can vary significantly, while the disparities in thermal conductivity and Seebeck coefficient may not be as pronounced as those observed in bulk materials. Analyzing the comprehensive impact of material composition on thermal conductivity, electrical conductivity, and the Seebeck coefficient can provide insights into the development of high-performance printed thermoelectric materials.

**Figure 7:**
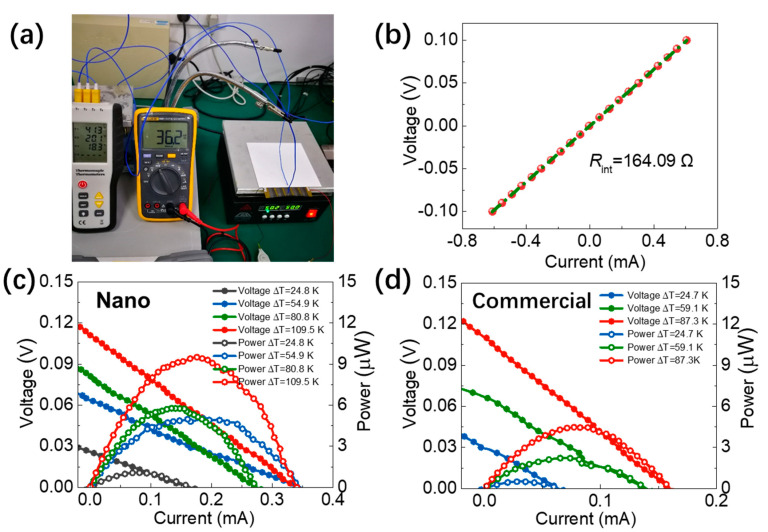
Electrical performance of the flexible TEG; (**a**) test environment and instrument; (**b**) internal resistance of the flexible TEG at room temperature; (**c**,**d**) relationship between thermal power and electrical output of the flexible TEG made of Bi_2_Se_3_ nanoflakes and commercial powders, respectively.

During the single-layer tests, four thermocouples were strategically positioned at the heat source, ambient, hot end, and cold end of the device to monitor the temperature variations. The temperature data was recorded and analyzed. The *I-V* characteristics of the device were scanned and measured using a semiconductor parameter analyzer (Keithley 4200-SCS). The corresponding results can be observed in Figure 7a and Supplementary Figure S2. The maximum power output, *P*_max_, of the generator can be achieved by matching the external load resistance, R_load_, with the internal resistance of the generator, *R*_int_, i.e., *R*_load_ = *R*_int_, and it is related to *V*_oc_, where *V*_oc_ is the open-circuit voltage of the generator and *I*_sc_ is the short-circuit current. The value of *P*_max_ depends on the *V*_oc_ and *R*_int_ of the generator. By optimizing the load resistance to match the internal resistance, the maximum power transfer can be achieved, as shown in the following equation:

(3) $$V_{\mathrm{oc}}=N\cdot \left(\alpha_{p}-\alpha_{n}\right)\cdot \Delta T$$

(4) $$P_{\max}=\frac{V_{\mathrm{oc}}{}^{2}}{4R_{\mathrm{int}}}$$

**Figure 8:**
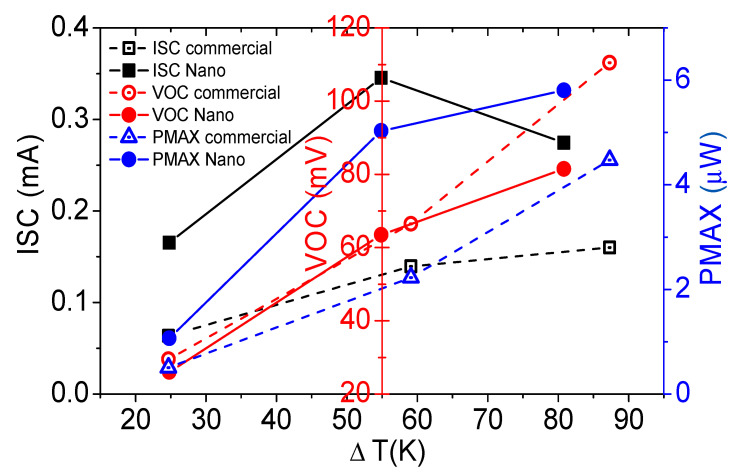
Output performance comparison of the two flexible TEGs made of Bi_2_Se_3_ nanoflakes and commercial powders.

When there is no temperature difference between the two sides of the device, the *I*-*V* characteristic of a flexible TEG is tested. The slope of Figure 7b in the static state represents the internal resistance of the device at room temperature. The internal resistance of the flexible TEG made of Bi_2_Se_3_ nanoflakes and Sb_3_Te_3_ is 164.09 Ω, much lower than the device made of commercial Bi_2_Se_3_ powder of 465.18 Ω. According to Equations (3) and (4), the flexible TEG made of synthetic nanoflakes has greater advantages. Figure 7c,d shows the relationship between thermal power and electrical output of the flexible TEG made of Bi_2_Se_3_ nanoflakes and commercial powders, respectively. When the temperature difference between the two ends of the nanodevice is 109.5 K, the open-circuit voltage is 0.11 V, the short-circuit current is 0.34 mA, the maximum output power is 9.5 μW, and the internal resistance fluctuates between 157 and 326 Ω. Each output performance exceeds the device prepared by commercial materials (Table 1 and Figure 7c,d). Due to the temperature dependence of the electrical conductivity of Bi_2_Se_3_ nanoflakes, there is a portion in the low-temperature range where the conductivity decreases with increasing temperature. This leads to an increase in the internal resistance of the device, resulting in a decrease in short-circuit current and causing non-parallel or even intersecting behavior in the IV curve. With a further increase in operating temperature, it can be observed that as the conductivity increases, the internal resistance decreases, leading to an increase in the short-circuit current and causing the curves to become more parallel again. The thermoelectromotive force of the device is generally 1.01 mV·K^−1^, which is distributed to a pair of TE legs as 200 μV·K^−1^. This value is slightly lower than the material properties. This is due to the internal transfer of heat due to the difference in PN thickness. The similar combinations article reported the use of the MWCNT hybrid networks technique to form thermoelectric composite materials with Bi_2_Se_3_ and Sb_2_Te_3_, which exhibited good electrical resistance stability even at bending radii of 5 mm or less [44,45]. Additionally, the combination of the PVA/MWCNT hybrid process was employed to fabricate thin-film thermoelectric devices with both inorganic thermoelectric materials. The planar TEGs demonstrated a maximum thermally generated voltage of 3 mV at ΔT = 20 K. Compared to our work, the reported study utilized the MWCNT process, which exhibited superior flexibility. In contrast, our approach in this article employed a higher filler ratio, leading to electrical characteristics that closely resemble those of inorganic semiconductors. Figure 8 also shows the comparison of the two flexible TEGs made of Bi_2_Se_3_ nanoflakes and commercial powders. The flexible TEG made of Bi_2_Se_3_ nanoflakes has a higher output powder and much thinner thickness compared with the commercial powder, and the output power can be increased by print multilayer or more TE legs, which is of great significance for the application of microflexible TE generators.

## 4. Conclusions

We used screen printing to prepare a microflexible TE material based on n-type Bi_2_Se_3_ nanoflakes and measured the Hall effect and Seebeck effect of the printed films. Combined with the TE legs prepared by p-type Sb_2_Te_3_ powder printing, a strip π-type TE device with five pairs of TE legs was formed. The flexible TE material based on Bi_2_Se_3_ nanoflakes had a conductivity of 75 S·cm^−1^ at room temperature, and the Seebeck coefficient was close to that of the bulk, which expands the choice of TE materials for flexible TE devices. The maximum performance of the nanodevice was obtained at a temperature difference of 109.5 K, the open-circuit voltage was 0.11 V, the short circuit current was 0.34 mA, and the maximum output power was 9.5 μW. Its performance exceeded that of devices made from commercial raw materials and is expected to provide an energy supply for flexible electronic devices.

## Figures and Tables

**Table 1:** TE-conversion electrical performance parameters of the two flexible TEGs.

	Δ*T* (K)	*I_sc_* (μA)	*V*_oc_ (mV)	*P*_max_ (μW)	*R*_int_ (Ω)	*E* (mV·K^−1^)	*Ρ* (mW·cm^−1^·K^−1^)
Bi_2_Se_3_ nanoflakes	24.8	165.27	26.02	1.06	157.44	1.05	0.0536
	54.9	345.37	63.51	5.03	183.87	1.16	0.1146
	80.8	274.36	81.51	5.80	297.07	1.01	0.0898
	109.5	340.43	111.00	9.51	326.07	1.01	0.1086
Commercial Bi_2_Se_3_	24.7	63.37	29.48	505.73	465.18	1.19	0.0256
	59.1	139.46	66.51	2.23	476.87	1.13	0.0471
	87.3	160.01	110.49	4.47	690.51	1.27	0.0640

## Data Availability

The data that support the findings of this study are available from the corresponding author upon reasonable request.

## Supplementary Materials

The following supporting information can be downloaded at: https://www.mdpi.com/article/10.3390/nano13131937/s1, Figure S1: Schematic illustrations and photographs of the samples; Figure S2: The flexible TEG and Equipment for testing the TEG; Figure S3: Performance of Sb_2_Te_3_; Figure S4: Performance of Sb_2_Te_3_; Table S1: Parameters of the flexible TEG.
