# Supplementary material for: Exploring Material Properties and Device Output Performance of a Miniaturized Flexible Thermoelectric Generator Using Scalable Synthesis of Bi2Se3 Nanoflakes
Source: Nanomaterials (Basel). 2023 Jun 26;13(13):1937. doi: 10.3390/nano13131937 (PMC10343555; doi:10.3390/nano13131937)
Supplement: Supplementary file 1 [file nanomaterials-13-01937-s001.zip › nanomaterials-2439441-supplementary.pdf]

Supporting Information

Scalable Synthesis of Bi_2Se_3 Nanoflakes for the Micro Flexible Thermoelectric Generator

Zicheng Yuan[1], Xueke Zhao[2], Canhui Wang[1], Shuang Hang[3], Mengyao Li[3*], Yu Liu[4*]

1.Reactor Engineering Sub-Institute, Nuclear Power Institute of China, 610213 Chengdu, P.R. China

2.School of Physics and Microelectronics, Zhengzhou University, 450052 Zhengzhou, P.R. China

3. Inter-university Institute for High Energies, Université Libre de Bruxelles, 1050 Brussels, Belgium

4.School of Chemistry and Chemical Engineering, Hefei University of Technology, 230009 Hefei, P.R. China

First author- yuanzc_npic@163.com

Corresponding author- limengyaorz@zzu.edu.cn; yliu@hfut.edu.cn

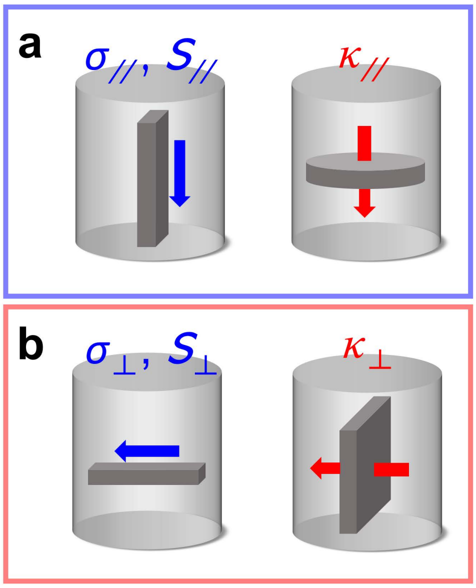

Figure S1. Schematic illustrations and photographs of the samples obtained from the cylindrical pellets and used to measure electrical conductivities (σ), Seebeck coefficients (S) and thermal conductivities (κ) in both directions, (a) parallel (//) and (b) normal ($\perp$) to the press direction.

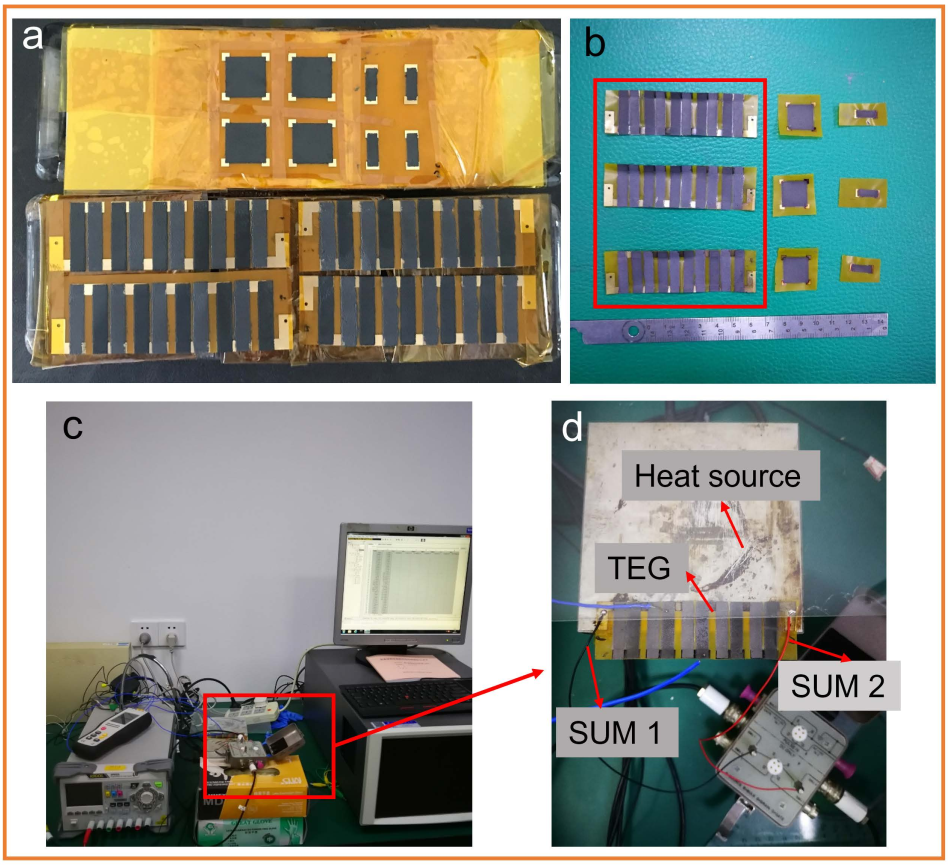

Figure S2. a, b) The flexible TEGs; c, d) Equipment for testing the TEGs.

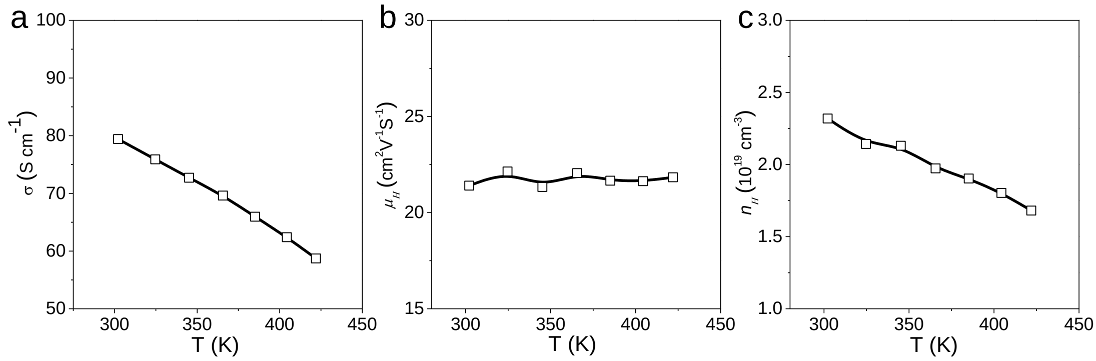

Figure S3. a) electrical conductivity, σ; b) mobility, μ_H; and c) carrier concentration, n_H of commercial Sb_2Te_3 powder.

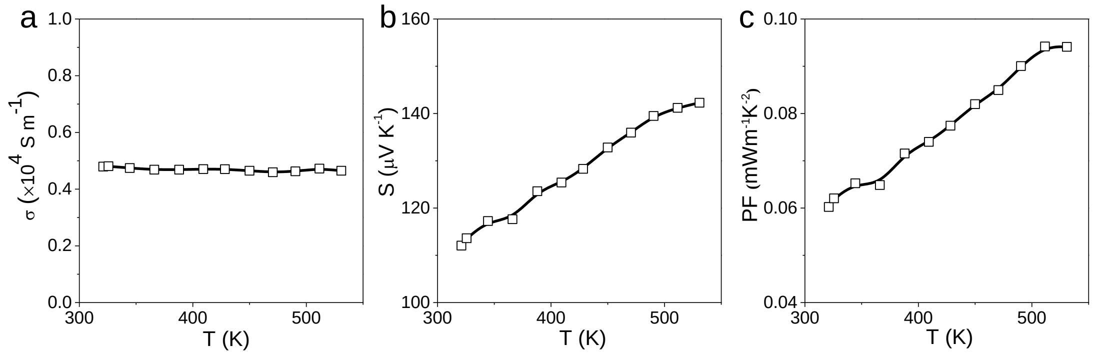

Figure S4. a) electrical conductivity, σ; b) Seebeck coefficient, S; and c) power factor, PF of commercial Sb_2Te_3 powder.

Table S1. Parameters of the flexible TEG.

	Length mm	Width mm	Thickness μm
substrate	87.5	25	50
p-type TE legs	25	5	60
n-type (nano)	25	5	8
n-type (commercial)	25	5	60
electrode	12.5	4	50
